# Spinal arachnoid cyst among Nigerians

**DOI:** 10.1093/jscr/rjaf331

**Published:** 2025-05-21

**Authors:** Toyin A Oyemolade, Augustine A Adeolu, Oluwakemi A Badejo, Olusola K Idowu, Ifeanyi E Iwuagwu

**Affiliations:** Department of Neurological Surgery, University College Hospital Ibadan, Queen Elizabeth Road, Oritamefa, PMB 5116, Ibadan, Oyo State, Nigeria; Division of Neurosurgery, Department of Surgery, Federal Medical Centre, Owo, Michael Adekunle Ajasin Road, PMB 1053, Owo, Ondo State, Nigeria; Department of Neurological Surgery, University College Hospital Ibadan, Queen Elizabeth Road, Oritamefa, PMB 5116, Ibadan, Oyo State, Nigeria; Division of Neurosurgery, Department of Surgery, College of Medicine, University of Ibadan, Queen Elizabeth Road, Oritamefa, PMB 5116, Ibadan, Oyo State, Nigeria; Department of Neurological Surgery, University College Hospital Ibadan, Queen Elizabeth Road, Oritamefa, PMB 5116, Ibadan, Oyo State, Nigeria; Division of Neurosurgery, Department of Surgery, Federal Medical Centre, Owo, Michael Adekunle Ajasin Road, PMB 1053, Owo, Ondo State, Nigeria; Department of Anaesthesia, College of Medicine, University of Ibadan, University College Hospital Ibadan, Queen Elizabeth Road, Oritamefa, PMB 5116, Ibadan, Oyo State, Nigeria; Department of Neurological Surgery, University College Hospital Ibadan, Queen Elizabeth Road, Oritamefa, PMB 5116, Ibadan, Oyo State, Nigeria

**Keywords:** arachnoid cyst, spinal, management, outcome, Nigeria

## Abstract

Spinal arachnoid cysts are rare spinal mass lesions. The pathogenesis is as yet poorly understood but the aetiology can be congenital or secondary to a number of infective and non-infective conditions. They can be extradural or intradural in location. They are characterized by a wide range of clinical manifestations, from asymptomatic lesions to severe myelopathy. Asymptomatic cysts can be managed conservatively while surgery is indicated in symptomatic cases. The operative technique of choice is complete excision of the cyst. The outcome is mainly related to the duration of symptoms, hence the need for early diagnosis and prompt treatment. In this study, we present the clinical profile and outcome of management of four operated cases of spinal arachnoid cysts in a tertiary hospital in Southwestern Nigeria.

## Introduction

Arachnoid cysts (ACs) are benign cranial or spinal cerebrospinal fluid containing mass lesions lined by normal or slightly thickened arachnoid membrane [1, 2]. Spinal arachnoid cysts (SACs) are relatively rare, accounting for 1%–3% of all mass lesions in the spinal canal [3]. The pathogenesis of SACs is not well understood but a number of theories have been described [3, 4]. Most cases are spontaneous but cases following trauma, infection, inflammation, intraspinal haemorrhage, lumbar puncture, laminectomy, and vertebroplasty have been reported [4–6]. They are commonly located in the thoracic region, may be dorsally or ventrally located in the spinal canal and can be extradural, intradural-extramedullary, or intramedullary [4, 7–9]. Nabors *et al*. classified SACs into type I—extradural arachnoid cysts (EACs) without spinal nerve root fibres (type Ia—EACs, type Ib—sacral meningoceles), type II—EACs with spinal nerve root fibres, and type III—intradural AC [10]. SACs may be asymptomatic or may present with motor, sensory, or autonomic symptoms due to compression of spinal cord and/or nerve roots [1, 9]. Increasing number of SACs are now being reported with wide spread use of magnetic resonance imaging (MRI) [9]. Symptomatic cases require surgical intervention, though the optimal surgical option remains controversial [4, 11]. There is paucity of data on these lesions among the local population. This study aims to evaluate the clinical profile and outcome of management of SACs in a tertiary hospital in Southwestern Nigeria.

## Case 1

**Figure 1:**
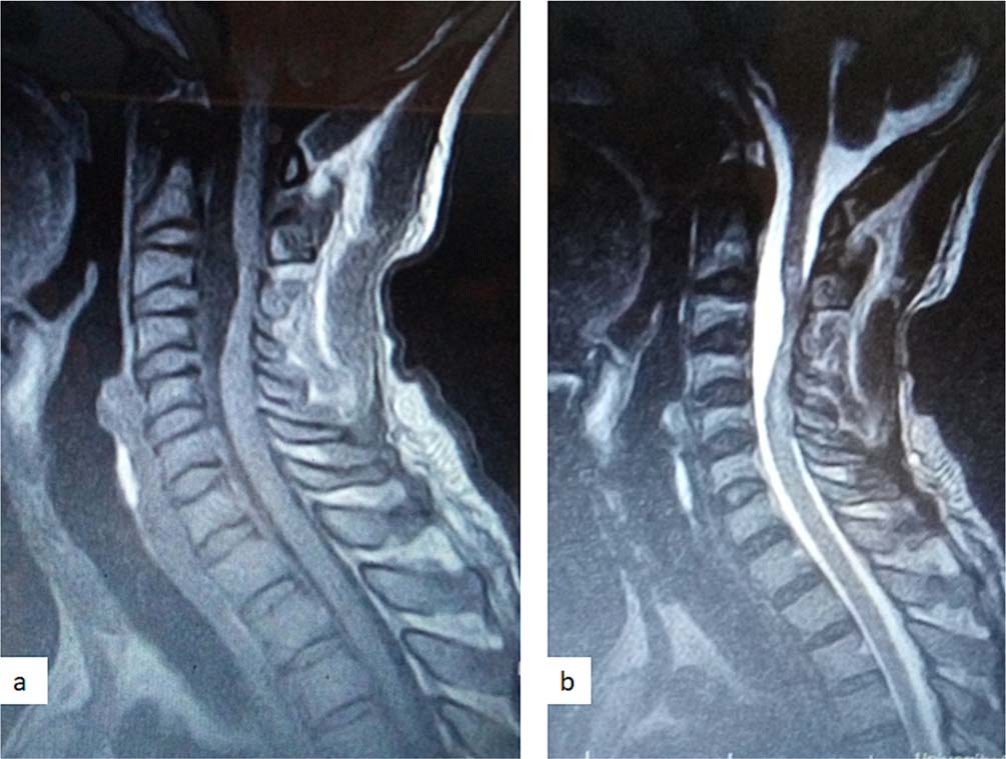
Sagittal T1-weighted (a) and T2-weighted (b) cervical spine MRI images showing an intradural arachnoid cyst posteriorly displacing and compressing the spinal cord.

**Figure 2:**
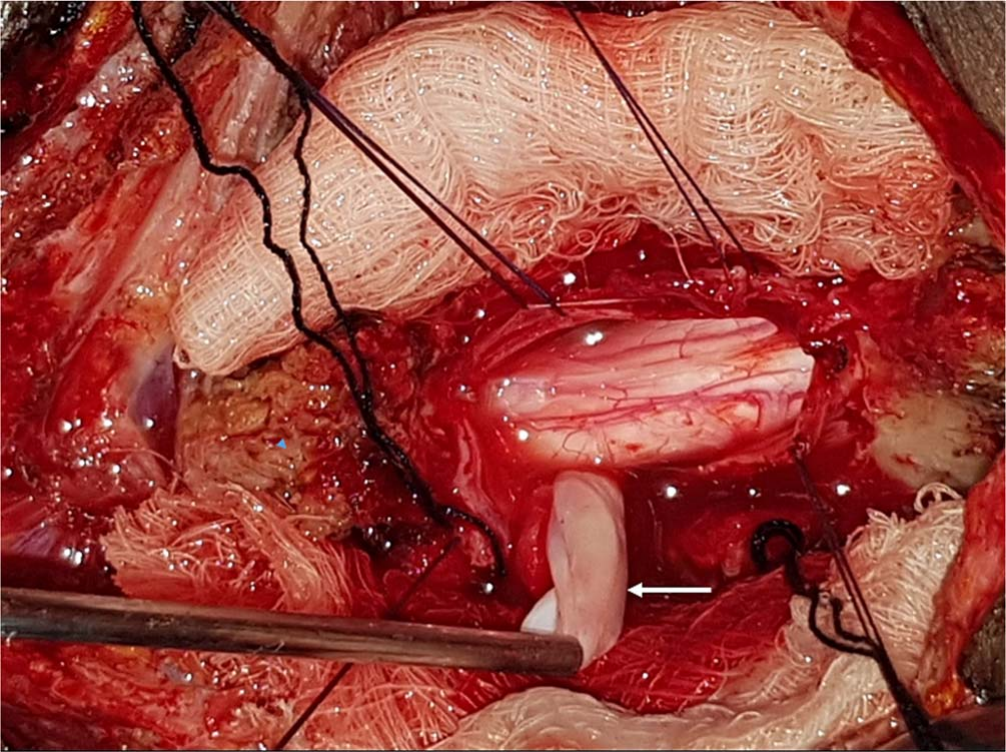
Intra-operative photograph showing complete excision of the arachnoid cyst in Fig. 1.

A 47-year-old man presented to us with progressive weakness of the extremities of 5 weeks duration. The weakness was first noticed in the left hand and progressed to involve the entire left upper limb and subsequently the right upper limb and the lower limbs. There was associated paraesthesia, constipation, and erectile dysfunction. Clinical examination revealed a middle-aged man with spastic quadriplegia and exaggerated muscle stretch reflexes. The sensory level was C4. Other systemic examination findings were normal. A clinical diagnosis of C4 non-traumatic myelopathy, Frankel B was made. Cervical spine MRI showed an intradural-extramedullary lesion with similar intensities to cerebrospinal fluid (CSF) on all sequences anterior to the spinal cord at C2–C4 with significant cord compression at C3–C4 and cord signal change on T2-weighted image at C2–C5 (Fig. 1). A diagnosis of cervical spine intradural AC was made. The lesion was accessed through C3–C4 laminectomies. At surgery, there was a cyst anterior to the cord with the latter flattened and displaced posteriorly. The cyst was excised completely and water tight dura closure done (Fig. 2). He made progressive post-operative neurological improvement and he was discharged on the 10th post-operative day. He was last seen 22 months post-surgery. At the time, he was ambulating without support, power was Grade 4+ to 5 in the upper and lower limbs.

## Case 2

**Figure 3:**
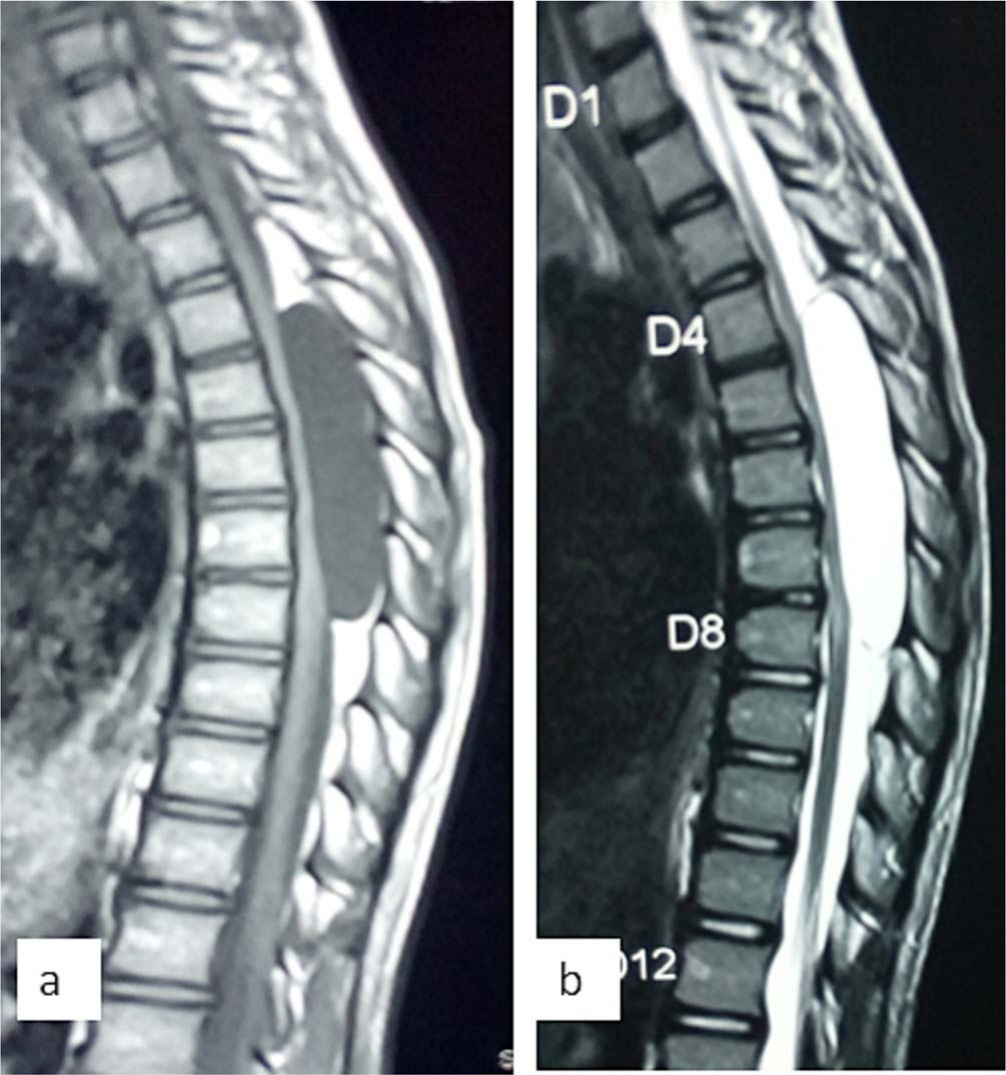
Sagittal T1-weighted (a) and T2-weighted (b) thoracic spine MRI showing an extradural cyst at T4–T8 compressing the cord anteriorly [12].

**Figure 4:**
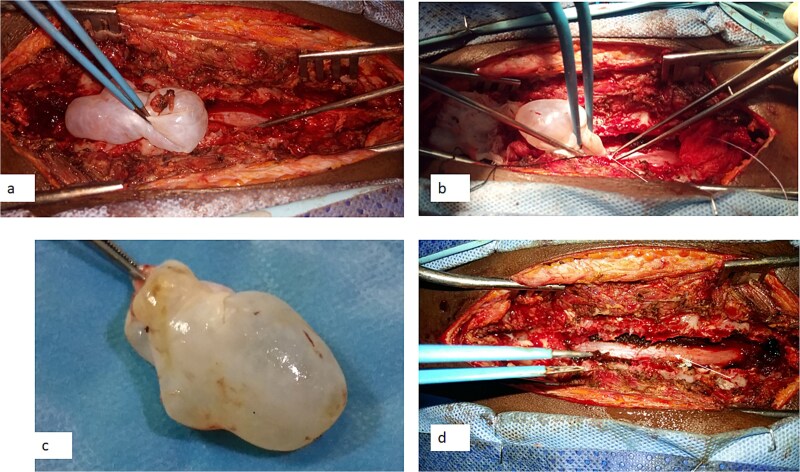
Intra-operative photograph showing complete excision of the arachnoid cyst in Fig. 3. (a) Dissection of the cyst from the dura, (b) the point of communication of the cyst with the subdural space (arrow) [12], (c) the excised cyst, and (d) the dura after the excision of the cyst (arrow).

A 12-year-old girl presented to us with progressive weakness of the lower limbs of 11 months duration. There was associated paraesthesia and spasms but no sphincteric dysfunction. Clinical examination revealed an otherwise healthy young girl with normal mental status. She had spastic paraparesis (power Grades 3 to 4) and exaggerated muscle stretch reflexes in the lower limbs. The sensory level was T4. There was no gibbus or spinal tenderness. Other systemic examination findings were normal. A clinical diagnosis of T4 non-traumatic myelopathy, Frankel D was made. Thoracic spine MRI showed an extradural lesion with similar intensities to CSF on all sequences posterior to the spinal cord at T4–T8 as well as significant cord compression at the same levels (Fig. 3) [12]. A diagnosis of thoracic spine EAC was made. We approached the lesion through T4–T8 laminectomies. Intra-operative finding was that of a large extradural cyst which communicated with the subarachnoid space through a dorsolaterally located pedicle near the nerve sleeve at T7. The cyst was excised completely and the dura defect closed without duroplasty (Fig. 4) [12]. The post-operative course was uneventful and she was discharged on the 24th post-operative day. She was last seen 48 months post-surgery. At the time, she was ambulating without support with power of Grade 5 in the lower limbs.

## Case 3

A 28-year-old man presented to us with progressive weakness of the lower extremities of 4-year duration. He had been non-ambulant in the 21 months preceding the presentation in our hospital. There was associated paraesthesia, urinary incontinence, constipation, and erectile dysfunction. Clinical examination revealed a young man with normal mental status. There was spastic paraparesis, power Grade 1–2 in the lower limbs, with exaggerated muscle stretch reflexes. The sensory level was T6. Other systemic examination findings were normal. A clinical diagnosis of T6 non-traumatic myelopathy, Frankel C was made. Thoracic spine MRI showed an intradural-extramedullary lesion with similar intensities to CSF on all sequences posterior to the spinal cord at T7–T9 with significant cord compression at the same levels and cord signal change on T2-weighted image at T8. A diagnosis of thoracic spine intradural-extramedullary arachnoid cyst was made. The cyst was approached posteriorly through T7–T9 laminectomies and midline durotomy. At surgery, there was a cyst posterior to a compressed and flattened spinal cord. The cyst was excised completely and water tight dura closure done. The post-operative course was uneventful but made no neurological gains. He was discharged on the 14th post-operative day. He remained neurologically the same at the time of last follow-up visit 17 months post-surgery.

## Case 4

A 34-year-old woman presented to us with progressive bilateral lower limbs weakness of 14 months duration. The symptom was gradual in onset and slowly progressive. She also complained of low back pain that radiates to both lower limbs. The latter preceded the limb weakness. Examination revealed a young woman with satisfactory general condition. Neurological examination of the upper limbs was normal. She had spastic paraparesis; power was Grade 0–2 in the left lower limb and 2–3 on the right. There was exaggerated deep tendon reflexes in the lower limbs with extensor plantar response. The sensory level was T10. A diagnosis of T10 non-traumatic myelopathy Frankel C with background lumbar spondylosis was made.

**Figure 5:**
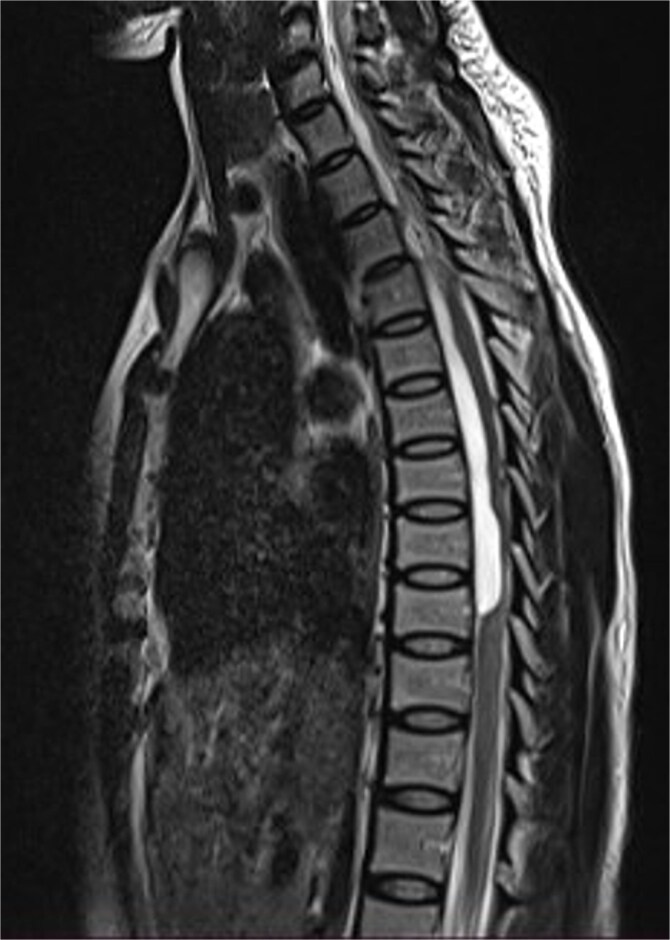
Sagittal T2-weighted thoracic spine MRI images showing an anteriorly located intradural arachnoid cyst at T5–T9, posteriorly displacing and compressing the spinal cord.

Thoracic and lumbosacral spine MRI showed an intradural-extramedullary cystic lesion, hypointense on T1 and hyperintense on T2-weighted images, extending from T5 to T9 vertebral levels with posterior displacement and flattening of the cord at the same levels (Fig. 5). There were multiple disc bulges at L3/L4, L4/L5, and L5/S1 with minimal thecal compression. A diagnosis of thoracic intradural AC was made.

The cyst was approached posteriorly through T5–T8 left hemilaminectomies. At surgery, there was a cyst anterior to and posteriorly displacing the spinal cord which was flattened. Partial cyst excision was done and water tight dura closure done. The post-operative course was uneventful, she remained neurologically the same and was discharged on the 17th post-operative day. Her first post-operative follow-up visit was 6 weeks after discharge (59 days post-surgery). At this time, she had not made any neurological gain.

## Discussion

SACs are uncommon cause of spinal cord and spinal nerve root compression with only few existing large series [3, 13]. Over a 30-year period, we operated on 3 adults and 1 paediatric patients with SACs, accounting for 3.2% of all operated cases of spinal tumours in our facility in keeping with the literature [3]. The cysts were located in the thoracic region in three patients and in the cervical region in the other. Three of the cases were intradural, while the other was extradural. All the patients presented with motor weakness. Total excision of the cyst was done in three of the cases and partial excision in the fourth patient. Two of the cases improved while the others remain neurologically the same at the time of last follow-up.

The reported gender predilection of SACs varies widely in the literature, with some reporting male predominance and others female. This variation may be because of rarity of SACs with a significant proportion of publications being on single cases or small series or may simply be because there exists no gender predilection [4, 13]. Our cases were equally distributed between males and females (Table 1). SACs have been reported in children and adults. In the adult population SACs are more common in the fifth and sixth decades of life [8, 14, 15]. One of our adult patients was in the fifth decade of life, while the other two were in the third and fourth decades.

**Table 1:** Clinical and demographic characteristics of the patients.

Serial number	Age (in years)	Sex	Symptoms	Duration of symptoms (in months)	Preoperative Frankel grade	Postoperative Frankel grade	Duration of follow-up (in months)
1	47	M	Pain and limb weakness	1	B	D	22
2	12	F	Limb weakness	11	D	E	48
3	28	M	Limb weakness	48	C	C	17
4	34	F	Pain and limb weakness	14	C	C	2

The pathogenesis of SACs is poorly understood. Congenital cases, as well as cases following surgery, infection, inflammation, and trauma have been reported [4–6]. Acquired aetiology could not be established in any of our four patients, we therefore presumed that they were all congenital.

SACs may be extradural or intradural. Most series reported extradural cysts to be more common than intradural cysts [3, 9]. This may however not be the case in children. In a large paediatric series, Bond *et al*. reported that 58% of their cases were intradural and proposed that this may be related to congenital abnormalities of the central nervous system (CNS), which are known to be associated with intradural cysts [13]. Intramedullary SACs are less frequently encountered compared to the others [9, 13]. SACs are predominantly located in the thoracic region and more commonly dorsal to the spinal cord. The thoracic predilection may be due to the thoracic spine being the longest spinal segment or the thoracic SACs being the most likely to be symptomatic because of the narrow thoracic spinal canal [9].Three of our cases were in the thoracic region while the other was cervical. The only paediatric case in this series was thoracic and extradural, while the three adult cases were intradural.

**Figure 6:**
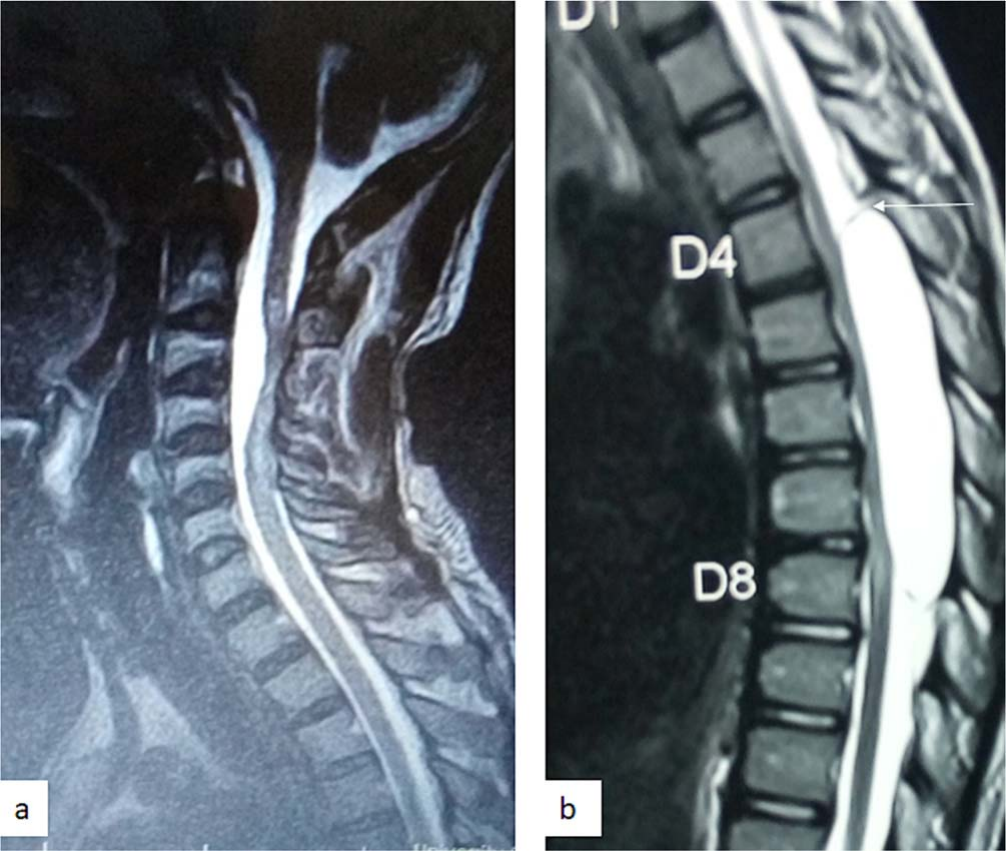
(a) Sagittal T2-weighted cervical spine MRI image showing expanded subarachnoid space anterior to the spinal cord at C2–C4, posterior displacement and compression of the cord and cord signal change at C2–C5. The wall of the cyst is indistinguishable suggestive of intradural spinal arachnoid cyst. (b) Sagittal T2-weighted thoracic MRI image showing compression of the spinal cord anteriorly. There is visible hypointense cyst wall (arrow) suggestive of extradural cyst.

MRI is the imaging modality of choice for the diagnosis and follow-up of SACs [4]. It does not require intrathecal injection of contrast and demonstrates the location, size, extent, and nature of the cysts as well as neural elements compression and intrinsic cord changes among other features [7, 16–18]. On MRI SACs have similar signal intensities to those of CSF, hypointense on T1-weighted, and hyperintense on T2-weighted images [16]. Extradural cysts may show absent posterior epidural fat, epidural fat capping, and T2-hypointense cyst wall [16, 19, 20] (Figs 3 and 6b). Intradural cysts are characterized by widening of the subarachnoid space, displacement of the cord/cord compression and an undistinguishable cyst wall [2, 21] (Fig. 1). The site of communication between the cysts and subarachnoid may not be demonstrable on MRI [16]. Computed tomography myelograghy has been the imaging of choice in demonstrating the communicating site between the cyst and the subarachnoid cyst [16]. Newer MRI flow studies using cinematic MRI has proven to also be able to demonstrate the communication site [22].

Asymptomatic SACs can be managed conservatively with observation and clinical and imaging follow-up [3, 12, 23]. Surgical intervention is indicated in symptomatic cases [3, 24, 25]. The surgical options include aspiration, disconnection from subarachnoid space, fenestration, shunting (cystopleural, cystoperitoneal, or cystosubarachnoid), partial or complete excision [3, 4]. The goal of surgery is neural decompression and prevention of cyst refilling [4, 26]. Complete cyst excision provides the least chance of recurrence and, therefore, is the treatment of choice when technically feasible [4, 8, 13]. In intradural cysts, fenestration of the cyst is less associated with reaccumulation and may be the preferred option of treatment in cases where complete excision may lead to undue injury to neural elements [3, 13]. Simple cyst aspiration is inadequate and, thus, not recommended because the cysts tend to refill [9, 19, 26, 27]. Shunting is reserved for recurrent/refractory cases [13, 24, 28]. The outcome of treatment depends mainly on the duration and the degree of neural compression with early surgical intervention offering the best chance of functional recovery [26, 29]. Complete excision was done in three of our patients while the last case, an intradural cyst, had partial excision of the cyst. Post-operative improvement was observed in the two patients with pre-operative duration of symptoms <1 year, while our patients with duration of symptoms more than a year remained neurologically the same at the time of last follow-up, albeit the patient with duration of symptoms of 14 months had only been followed up for 2 months (Table 1).

## Conclusion

SACs are rare spinal causes of neurological dysfunction with yet to be fully understood aetiopathogenesis. They are more frequently thoracic in location and posterior to the spinal cord. Symptomatic cases require surgical intervention. Complete surgical excision is the treatment of choice. Outcome of treatment appears to be mainly determined by the duration of symptoms and the degree of neural compression. Prompt diagnosis and early surgical intervention offer the best chance of neurological recovery.
